# Colorimetric Detection of the *vanA* Gene in Vancomycin-Resistant Enterococcus Using Loop-Mediated Isothermal Amplification with Hydroxy Naphthol Blue

**DOI:** 10.3390/ijms27219927

**Published:** 2026-11-05

**Authors:** Sawanya Pongparit, Kantima Choosang, Bajaree Jantrapanukorn, Rungnapa Veeramano, Suphitcha Augkarawaritsawong, Khurawan Kumkrong, Parthompong Ves-urai, Pongbun Tangjitrungrot, Naiyana Wattanakul, Kosum Chansiri, Saranthum Phurijaruyangkun

**Affiliations:** 1 Faculty of Medical Technology, Rangsit University, Pathum Thani 12000, Thailand; sawanya.p@rsu.ac.th (S.P.); kantima.c@rsu.ac.th (K.C.); bajaree.j@rsu.ac.th (B.J.); rungnapa.v@rsu.ac.th (R.V.); suphitcha.a@rsu.ac.th (S.A.); khurawan.k@rsu.ac.th (K.K.); parthompong.v@rsu.ac.th (P.V.-u.); 2 Center of Excellence in Biosensors, Panyananthaphikhu Chonprathan Medical Center, Srinakharinwirot University, Nonthaburi 11120, Thailand; pongbun@g.swu.ac.th; 3 Nopparatrajathanee Hospital, Bangkok 10230, Thailand; naiya447@gmail.com; 4 Strategic Wisdom and Research Institute, Srinakharinwirot University, Bangkok 10110, Thailand; kosum@g.swu.ac.th

**Keywords:** *vanA*, vancomycin-resistant Enterococcus, loop-mediated isothermal amplification, hydroxy naphthol blue, colorimetric detection

## Abstract

Vancomycin-resistant enterococci (VRE), particularly vancomycin-resistant *Enterococcus faecium*, are important healthcare-associated pathogens, and *vanA* is a major acquired determinant of high-level glycopeptide resistance. The conventional polymerase chain reaction (PCR) method is reliable for molecular detection of *vanA* but requires thermal cycling and, in conventional formats, post-amplification analysis. In this study, we developed and evaluated a closed-tube colorimetric loop-mediated isothermal amplification assay using hydroxy naphthol blue (LAMP-HNB) for visual detection of *vanA.* A total of 130 Enterococcus isolates were tested, comprising 100 previously characterized in our published LAMP–lateral flow dipstick (LAMP-LFD) study (52 *vanA*-positive and 48 *vanA*-negative) and an additional 30 (13 *vanA*-positive and 17 *vanA*-negative), yielding 65 PCR-positive and 65 PCR-negative isolates overall. Reaction conditions were optimized sequentially, and the final LAMP-HNB protocol took place at 63 °C for 45 min with 4.0 mM MgSO_4_ and 5 μM HNB. The visual endpoint was confirmed by 2% agarose gel electrophoresis. LAMP-HNB correctly classified all 65 PCR-positive and all 65 PCR-negative isolates, yielding 100% sensitivity (65/65; exact 95% CI: 94.48–100%), 100% specificity (65/65; 95% CI: 94.48–100%), and 100% overall agreement (130/130; 95% CI: 97.20–100%). The limit of detection was 8.67 × 10^3^ genome copies/µL (28.52 pg/µL) for LAMP-HNB compared with 8.67 × 10^4^ genome copies/µL (285.2 pg/µL) for conventional PCR, representing a 10-fold lower DNA concentration endpoint under the experimental conditions employed. In the 100-isolate overlapping cohort, LAMP-HNB, the previously reported LAMP-LFD platform, and PCR were concordant. With its 45 min requirement for amplification after DNA preparation, the HNB format provides a rapid, closed-tube visual endpoint without a labeled probe, lateral flow strip, running buffer, or post-amplification hybridization step. These findings support LAMP-HNB as a promising molecular screening approach for *vanA*-positive enterococci, while larger, fully independent multicenter validation remains necessary before routine implementation.

## 1. Introduction

Enterococci illustrate a recurring problem in antimicrobial resistance: organisms that normally colonize the host can become difficult-to-treat pathogens after accumulating resistance and adapting to healthcare environments. *Enterococcus faecalis* and *Enterococcus faecium* account for most clinically important enterococcal infections, including urinary tract infection, bacteremia, endocarditis, intra-abdominal infection, and device-associated disease [1,2,3,4]. The concern is especially pronounced for *E. faecium* because hospital-associated lineages frequently carry multiple acquired resistance determinants. Reflecting this burden, the World Health Organization placed vancomycin-resistant *E. faecium* in the 2024 Bacterial Priority Pathogens List high-priority group [5].

*VanA*-mediated resistance results from replacing the terminal D-Ala-D-Ala target with D-Ala-D-Lac, markedly reducing glycopeptide binding [6,7,8,9,10]. Because *vanA* is a major determinant of high-level transferable vancomycin resistance, its rapid detection can complement phenotypic antimicrobial susceptibility testing and support timely resistant enterococci characterization [11].

Conventional PCR is widely used for *vanA* detection but requires thermal cycling and post-amplification analysis. Loop-mediated isothermal amplification (LAMP) provides an alternative approach in which target DNA is amplified at a constant temperature using a strand-displacing polymerase and multiple primers [12,13], with its high product yield also permitting several simple endpoint detection strategies [14,15,16].

Hydroxy naphthol blue (HNB) is a metal-ion indicator that can be incorporated into a reaction before amplification. Changes in free Mg^2+^ associated with LAMP product formation produce a visible color transition, allowing closed-tube interpretation and reducing post-amplification handling [17,18,19]. HNB-based *vanA* LAMP has previously demonstrated the feasibility of visual detection in Enterococcus isolates [20,21]. HNB was chosen for its magnesium-dependent response and compatibility with a pre-added, closed-tube endpoint; unlike pH-sensitive dyes, its readout does not primarily depend on a pH shift. In this study, we did not directly compare HNB with pH-dependent or other visual indicators, and no claim of superior visual discrimination is made.

In our previous study, we evaluated a *vanA*-targeted LAMP assay with a lateral flow dipstick (LAMP-LFD) using 100 Enterococcus isolates and demonstrated complete agreement with conventional PCR [22]. The LFD format provides a clear strip-based endpoint but requires post-amplification hybridization and strip handling. In the present study, we therefore evaluate HNB as a simpler closed-tube endpoint using the same primer set and analytical specificity panel, and 100 of the 130 isolates overlap. The present comparison of HNB and LFD is operational and based on separate study workflows, not a prospectively randomized or fully paired head-to-head comparison.

The LAMP-HNB assay was assessed using 130 non-duplicate Enterococcus isolates: 100 from the previously characterized LAMP-LFD cohort and an additional 30. The objectives were to determine agreement with conventional PCR, establish analytical sensitivity and specificity, and compare the operational characteristics of HNB detection with the previously reported LFD platform.

## 2. Results

### 2.1. Study Isolate Composition and Cohort Structure

The LAMP-HNB evaluation comprised 130 non-duplicate Enterococcus isolates with a prespecified two-part structure (Table 1). The overlapping set contained 100 isolates from the previously reported LAMP-LFD study, including 52 *vanA*-positive and 48 *vanA*-negative isolates assessed by conventional PCR [22], while the additional set included 30 that were not included in that publication: 13 *vanA*-positive and 17 *vanA*-negative. Accordingly, the combined analysis included 65 PCR-positive and 65 PCR-negative isolates. Results are presented separately for the two sets before the combined analysis to distinguish paired re-evaluation of the earlier isolates from observations in the additional collection.

### 2.2. Optimization of LAMP-HNB Reaction Conditions

The *vanA*-targeted LAMP-HNB reaction was optimized sequentially for incubation temperature, MgSO_4_ and HNB concentration, and reaction time. Temperatures of 61, 62, 63, 64, and 65 °C; MgSO_4_ concentrations from 3.0 to 5.5 mM; HNB concentrations of 1.0, 2.5, 5.0, 7.5, and 10 μM; and reaction times of 15, 20, 25, 30, 45, and 60 min were evaluated. Colorimetric endpoints were assessed by direct visual inspection and confirmed by 2% agarose gel electrophoresis (AGE). Positive reactions were characterized by a violet-to-blue transition accompanied by the characteristic ladder-like LAMP pattern on AGE, whereas negative and no-template controls remained violet and showed no specific amplification.

Among the conditions tested, 63 °C provided the most suitable incubation temperature. A final MgSO_4_ concentration of 4.0 mM supported clear amplification and visual discrimination, while 5 μM HNB produced the clearest positive-versus-negative color contrast without apparent amplification inhibition. During reaction time optimization, a clearly distinguishable positive endpoint was obtained at 45 min, though extending incubation to 60 min did not produce an appreciable improvement in the visual endpoint. The corresponding products at 45 and 60 min showed the expected ladder-like LAMP pattern on 2% AGE, confirming that the HNB color change reflected successful amplification.

The final optimized LAMP-HNB conditions were amplified at 63 °C for 45 min, with 4.0 mM MgSO_4_ and 5 μM HNB. The reaction time was selected as it was the shortest evaluated incubation period that provided a clear endpoint comparable to that observed at 60 min. These conditions were used for subsequent analytical specificity, analytical sensitivity, and evaluation of the 130 clinical Enterococcus isolates (Table 2).

### 2.3. Visual Detection by LAMP-HNB

**Figure 1:**
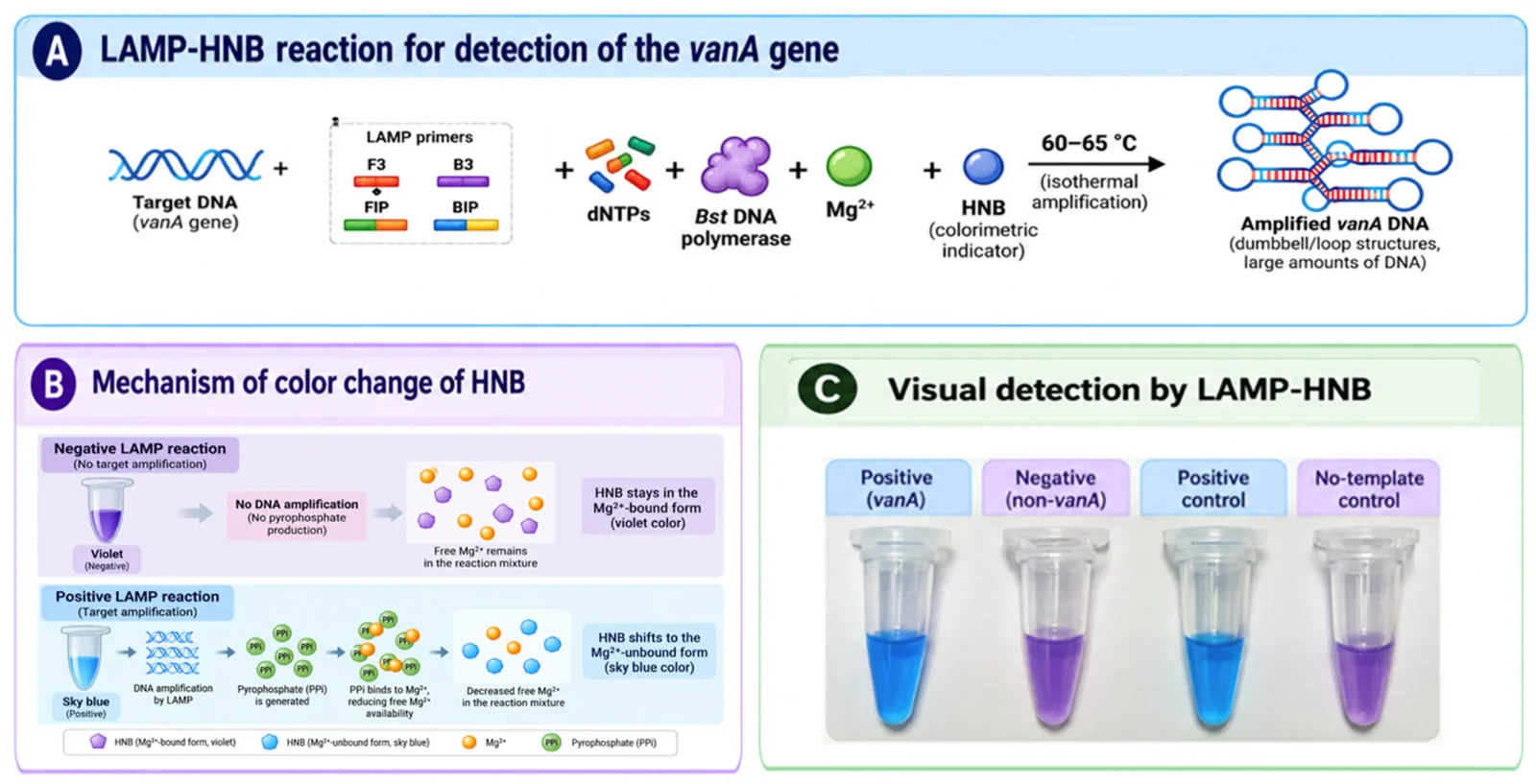
Visual detection by LAMP-HNB. (**A**) Schematic illustration of the LAMP-HNB reaction for *vanA* gene detection. The reaction mixture contains target DNA, primers (F3, B3, FIP, BIP), dNTPs, *Bst* DNA polymerase, Mg^2+^, and HNB, and is incubated under isothermal conditions. (**B**) Mechanistically, DNA polymerization releases pyrophosphate, which associates with magnesium to form magnesium pyrophosphate and reduces free Mg^2+^ availability. The resulting change in HNB metal-ion binding underlies the violet-to-blue color transition. This mechanism should also be reflected in the schematic artwork of the HNB colorimetric endpoint. (**C**) Representative LAMP-HNB results. *vanA*-positive reactions containing amplified *vanA* DNA exhibit a visible color change from violet to blue, whereas *vanA*-negative reactions retain their original violet color. The positive and no-template controls showed the expected positive and negative results, respectively. Because HNB was incorporated into the reaction mixture before amplification, the results could be interpreted directly in the closed reaction tubes without post-amplification manipulation.

HNB provided a visually distinguishable endpoint after amplification: *vanA*-positive reactions changed from violet to blue, whereas negative reactions retained their original violet appearance. Because HNB was added before incubation, the result was read directly in the closed reaction tube. Positive and no-template controls produced the expected outcomes, with representative colorimetric reactions shown in Figure 1. Visual readings were interpreted against the concurrently tested *vanA*-positive control (blue) and no-template control (violet), while weak or intermediate colors were not assigned a validated separate classification in this study; such ambiguous endpoints should not be treated as definitively positive or negative without confirmatory testing. The visual classification rules have not been independently validated for inter-reader agreement.

### 2.4. Agreement Between LAMP-HNB and Conventional PCR

In the overlapping set, LAMP-HNB reproduced the conventional PCR classification of all 100 isolates (52 positive and 48 negative). The same pattern was observed in the additional set, in which all 13 PCR-positive and all 17 PCR-negative isolates were correctly classified. The combined 2 × 2 analysis therefore contained 65 true-positive and 65 true-negative results, with none being discordant (Table 3).

### 2.5. Diagnostic Performance Relative to PCR

Using conventional PCR as the molecular comparator for *vanA*, the observed sensitivity and specificity of LAMP-HNB were each 100% (65/65; exact 95% CI: 94.48–100%), and overall agreement was 100% (130/130; 95% CI: 97.20–100%) (Table 4). Cohen’s kappa was 1.00, and PPV and NPV were also 100% in this balanced study set; these predictive values are descriptive of the evaluated collection rather than estimates for populations with differing *vanA* prevalence. The confidence intervals are retained because the zero observed errors do not establish error-free performance outside the tested sample.

### 2.6. Analytical Detection Limit

**Figure 2:**
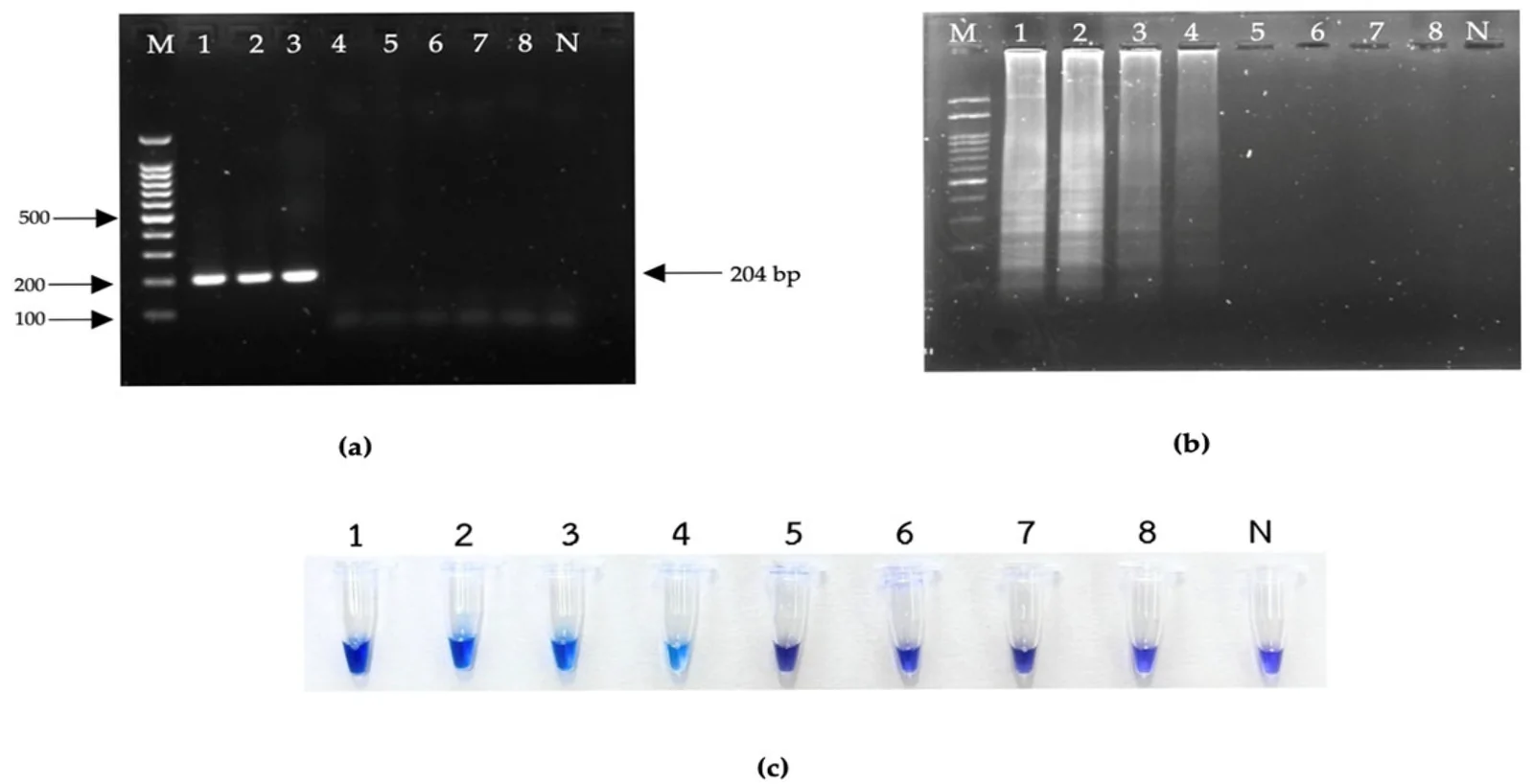
LAMP-HNB and conventional PCR analytical detection limits for *vanA*. Tenfold serial dilutions of genomic DNA from *vanA*-positive *Enterococcus faecium* ATCC 700221 were tested by LAMP-HNB and conventional PCR, based on an estimated bacterial genome size of 3.0 Mb. DNA concentrations corresponded to the following approximate genome copies/µL: 1: 8.67 × 10^6^ copies/µL; 2: 8.67 × 10^5^ copies/µL; 3: 8.67 × 10^4^ copies/µL; 4: 8.67 × 10^3^ copies/µL; 5: 8.67 × 10^2^ copies/µL; 6: 8.67 × 10^1^ copies/µL; 7: 8.67 copies/µL; 8: 8.67 × 10^−1^ copies/µL. N: No-template controls. (**a**) Conventional PCR with agarose gel electrophoresis (PCR-AGE). (**b**) LAMP with agarose gel electrophoresis (LAMP-AGE). (**c**) LAMP combined with hydroxy naphthol blue (LAMP-HNB). LAMP-HNB consistently detected *vanA* DNA down to 8.67 × 10^3^ copies/µL, whereas conventional PCR had a detection endpoint of 8.67 × 10^4^ copies/µ. Each concentration was evaluated in three independent runs. Under the experimental conditions used, LAMP-HNB therefore demonstrated a detection endpoint corresponding to a 10-fold lower DNA concentration than conventional PCR. The panels show representative results from the dilution experiments; each dilution is evaluated in three independent runs, but not all replicate images are displayed. The 285.18 ng/μL value is equivalent to approximately 8.67 × 10^3^ genome copies/µL and refers to the undiluted starting DNA stock; it is not shown as a numbered lane in this figure.

The LAMP-HNB assay’s analytical sensitivity was evaluated using tenfold serial dilutions of genomic DNA ranging from 28.52 ng/µL to 2.852 fg/µL. Based on an estimated bacterial genome size of 3.0 Mb, these concentrations corresponded to approximately 8.67 × 10^6^ to 0.867 genome copies/µL (Table 5). Across three independent runs, LAMP-HNB consistently detected *vanA*-positive genomic DNA; the limit of detection (LOD) of the assay was determined to be equivalent to approximately 8.67 × 10^3^ genome copies/µL or 1.73 × 10^4^ genome copies per reaction, whereas the endpoint for the conventional PCR protocol was equivalent to approximately 8.67 × 10^4^ genome copies/µL, assuming a DNA template volume of 2 µL; thus, under the experimental conditions used, the LAMP-HNB endpoint corresponded to a 10-fold lower DNA concentration (Figure 2). This comparison showcases the analytical dilution endpoints of the two protocols and is not used to infer a 10-fold difference in clinical sensitivity.

### 2.7. Paired Comparison with the Previously Reported LAMP-LFD Platform

The 100 Enterococcus isolates shared with the earlier study allowed a within-isolate comparison of endpoint formats [22]. Conventional PCR, the previously reported LAMP-LFD assay, and LAMP-HNB assigned the same 52 positive and 48 negative classifications in this set (Table 6). Consequently, these data do not demonstrate superior diagnostic accuracy of HNB over LFD; rather, they show that replacing the LFD endpoint with HNB did not alter classification in the isolates tested. The distinction between the platforms is principally procedural: HNB is incorporated before amplification and read in a closed tube, whereas LFD adds probe/strip reagents and post-amplification handling but provides a line-based readout with an internal control line. The 30 additional isolates were not used for the historical LFD comparison and were concordant only between LAMP-HNB and conventional PCR (13/13 positive and 17/17 negative).

## 3. Discussion

LAMP-HNB classified all 130 Enterococcus isolates in agreement with conventional PCR findings for *vanA*, with no false-positive or -negative results in the evaluated collection. The point estimates for sensitivity, specificity, and agreement were therefore 100%, but the lower confidence limits (94.48% for sensitivity and specificity; 97.20% for overall agreement) were more informative than the point estimates alone. They quantify the uncertainty that remains when no errors are observed in a finite sample. Accordingly, the present data support strong agreement with conventional PCR within this study rather than a claim of universally perfect diagnostic performance.

A central feature of this study is the isolate collection composition. The 100 Enterococcus isolates shared with our earlier LAMP-LFD investigation are not an independent validation cohort; their value lies in that the same molecularly characterized isolates can be used to examine whether changing the endpoint from LFD to HNB changes assay classification [22]. It does not: all 52 positive and 48 negative classifications are retained. By contrast, the 30 additional isolates are not represented in the earlier publication and provide a modest extension beyond the original collection, with concordant results in 13 positive and 17 negative isolates. This two-part interpretation is more appropriate than treating all 130 isolates as a single independent validation series.

The absence of discordance in the additional set is encouraging, but its size limits the strength of external validation claims. In particular, 13 additional positive isolates cannot represent the full genomic diversity of *vanA*-bearing enterococci, and 17 additional negative isolates cannot capture all potentially interfering genotypes or phenotypes encountered in routine practice. A subsequent study should therefore prioritize isolates assembled from multiple centers prospectively, include a broader distribution of species and vancomycin MICs, and prespecify challenging or discordant phenotypes. Such a design would test transportability rather than repeat agreement in a highly characterized collection.

Conventional PCR is used as the molecular comparator because both assays interrogate the presence of *vanA*. This distinction matters clinically: *vanA* status and phenotypic vancomycin susceptibility are related but not interchangeable. Other resistance determinants, particularly *vanB*, can produce vancomycin resistance without *vanA*, while unusual genotype–phenotype discordance has also been described. The HNB assay should therefore be positioned as a rapid *vanA*-targeted molecular test that complements, rather than replaces, organism identification and antimicrobial susceptibility testing. Because the conventional PCR comparator used the LAMP outer primers F3/B3 and was an in-house assay rather than an independently validated commercial molecular test, the observed concordance cannot establish accuracy against an independent clinical reference standard. Use of a validated commercial assay would strengthen future comparisons.

The analytical dilution experiment provides evidence that the LAMP-HNB protocol can detect lower template concentrations than the conventional PCR protocol used here; its endpoint of 8.67 × 10^3^ genome copies/µL is one dilution below the 8.67 × 10^4^ genome copies/µL PCR endpoint, corresponding to a 10-fold lower DNA concentration. A previous *vanA* LAMP study also demonstrated naked-eye HNB detection and reported greater analytical sensitivity than its PCR comparator [20]. Nevertheless, analytical LOD is protocol-dependent: extraction, input volume, primer chemistry, polymerase, endpoint definition, and replicate number can all shift the threshold. Therefore, we interpret the present result as a head-to-head comparison under the specified experimental conditions, not as a general property of LAMP relative to PCR. A prior publication by Kim et al. (2014) is specifically relevant to visualizing *vanA*-targeted LAMP with HNB and should be discussed as a prior achievement rather than presented as an innovation of this work [20]. Its experimental methods and quantitative performance should be checked against the original article before adding numerical comparisons.

Sequential optimization established the final reaction conditions as 63 °C for 45 min with 4.0 mM MgSO_4_ and 5 μM HNB. Importantly, the 45 min endpoint was visually clear and electrophoretically confirmed, while extending incubation to 60 min did not appreciably improve the endpoint. Selecting 45 min therefore shortens the amplification stage without sacrificing the observed qualitative readout under the tested conditions and strengthens the practical rationale for rapid *vanA* detection. The MgSO_4_ concentration used in the present LAMP-HNB assay (4.0 mM) was lower than that employed in our previous LAMP-LFD assay (5.0 mM), reflecting the different detection principles of the two methods. In the LAMP-HNB assay, hydroxy naphthol blue acts as a metal-ion indicator that responds to changes in free Mg^2+^ concentration during DNA amplification. Pyrophosphate generation during LAMP leads to magnesium pyrophosphate formation, thereby reducing the availability of free Mg^2+^ and inducing a color transition from violet to sky blue [15,17]. Consequently, an excessive concentration of MgSO_4_ may maintain higher residual free Mg^2+^ levels, potentially reducing the visual contrast between positive and negative reactions. In contrast, the LAMP-LFD assay relies on detecting labeled amplification products rather than Mg^2+^-dependent colorimetric changes. Therefore, the use of 4.0 mM MgSO_4_ in the present assay was intended to provide an appropriate balance between DNA amplification and HNB-based visual detection.

Previous studies, including Kim et al. (2014), have demonstrated the feasibility of naked-eye detection of the *vanA* gene using HNB-based LAMP, indicating that the underlying colorimetric detection principle is not novel [20]. Therefore, the principal contribution of the present study lies in the additional evaluation of this detection approach rather than the development of a novel *vanA* primer design or the first demonstration of HNB-mediated *vanA* detection. Specifically, the LAMP-HNB assay was evaluated using 130 clinical Enterococcus isolates and demonstrated complete agreement with conventional PCR (130/130 isolates). A comparison of the analytical performance and methodological characteristics of the present assay with previously published *vanA*-targeted LAMP methods is presented in Table 7. These findings provide additional evidence supporting the analytical performance and practical applicability of HNB-based LAMP detection in the clinical isolate panel examined. Nevertheless, differences in sample preparation, DNA input, amplification conditions, and definitions of detection limits restrict direct quantitative comparisons of analytical sensitivity across studies. Accordingly, the present work should be regarded as an incremental methodological evaluation that contributes further evidence on the applicability of LAMP-HNB for *vanA* detection rather than the introduction of a fundamentally new assay principle.

The practical rationale for HNB is strongest at the detection step; HNB is present before amplification and converts the 45 min amplification protocol into a closed-tube visual assay, avoiding the probe hybridization, strip, running buffer, and handling steps required by the previously evaluated LFD workflow [17,22]. Fewer post-amplification manipulations may shorten hands-on processing and reduce opportunities for amplicon carryover. HNB also removes the need for labeled primers/probes and disposable strips. These advantages are operational, however, and should not be conflated with greater target specificity: HNB is an indirect indicator of amplification-associated changes in free Mg^2+^, whereas LFD uses labeled amplicons and probe-mediated capture to generate a test line.

The LFD format therefore retains features that may be preferable in some settings. A discrete test line can be easier to score than a color transition, and the control line provides an immediate indicator of strip migration and reagent function. HNB interpretation may be influenced by illumination, tube material, observer perception, and weak reactions near the detection threshold. Standardized lighting blinded multi-reader assessment, or smartphone-based color quantification could strengthen future evaluation of readout reproducibility. Conversely, PCR remains advantageous where thermal cyclers, electrophoresis or real-time detection, established quality systems, and multiplexing capability are available; its infrastructure requirements are the principal reason an isothermal closed-tube alternative may be useful in decentralized or resource-constrained laboratories. HNB also differs from pH-based readouts in the chemical variable monitored (free magnesium rather than pH); however, no comparative experiment in this study establishes which indicator is more robust. Intermediate endpoints should not be interpreted as unequivocal positives or negatives without an independently validated decision rule.

Analytical specificity further supports the primer system within the tested panel. No LAMP-HNB cross-reaction was observed with the *vanA*-negative organisms examined, including *vanB*-harboring *E. faecalis* ATCC 51299, whose inclusion is particularly relevant because it challenges the assay with an alternative clinically important glycopeptide-resistance genotype rather than only unrelated bacterial species. Even so, a 12-strain panel cannot exclude cross-reactivity across the full diversity of clinical and environmental sequences; in silico surveillance of primer-binding regions and testing of additional van genotypes would strengthen future validation.

Several limitations therefore define the appropriate scope of the conclusions. This study was conducted at a single center; most of the dataset consisted of previously characterized isolates; the newly added set was small; and the collection was deliberately balanced rather than prevalence based. PPV and NPV from this design should not be transferred directly to routine populations. In addition, visual HNB interpretation was not evaluated across multiple blinded readers, and the LOD was based on triplicate dilution endpoints rather than a larger replicate series used to estimate a probabilistic LOD95. These limitations do not negate the observed agreement, but they identify the experiments needed to move from analytical and retrospective evaluation toward clinical implementation. The three-replicate, tenfold-dilution endpoint is not an LOD95; estimating an LOD95 would require finer dilutions near the threshold and substantially more replicates (for example, 20–30 per concentration). Such experiments were not performed. Likewise, inter-reader reproducibility was not assessed in a blinded multi-observer study, and commercial PCR confirmation was not performed. The HNB–LFD comparison was therefore descriptive rather than a direct paired analytical superiority study.

First, the analytical detection limit was determined using tenfold serial dilutions tested in triplicate. The lowest concentration consistently detected in all three reactions (28.52 pg/μL, equivalent to approximately 8.67 × 10^3^ genome copies/µL) therefore represents an endpoint detection limit, not a statistically estimated LOD95. More closely spaced dilutions near this preliminary threshold, together with a larger replicate series (e.g., 20–30 reactions per concentration), would be needed to characterize detection probability more precisely. These additional experiments were not performed in the present study.

Second, the HNB-based colorimetric endpoint was evaluated by visual inspection without assessment by multiple independent blinded readers or objective spectrophotometric measurements. Although distinct sky-blue and violet colors were used to identify positive and negative reactions, respectively, the potential effects of observer variability, lighting conditions, and subtle or intermediate color transitions were not systematically investigated. Therefore, inter-reader reproducibility and standardized criteria for interpreting ambiguous color changes remain to be established. Future studies should incorporate blinded assessments by multiple independent observers, standardized color references, and quantitative colorimetric analysis to improve the objectivity and reproducibility of result interpretation. In particular, spectrophotometric measurement of optical density (OD) at 650 nm, as previously described by Goto et al. (2009), could provide an additional objective approach for distinguishing positive and negative LAMP-HNB reactions [17]. However, assay-specific absorbance thresholds would require experimental validation before routine implementation.

In summary, the HNB format does not contribute to an improvement in classification over the earlier LFD assay within the overlapping isolates; both endpoints are concordant with PCR. Its potential value is a simpler closed-tube workflow that preserves the observed *vanA* classifications while reducing post-amplification processing. The additional 30 isolates provide supportive evidence beyond the original cohort, but a fully independent multicenter study is the appropriate next step to establish reproducibility, clinical accuracy, and operational performance under routine laboratory conditions.

## 4. Materials and Methods

### 4.1. Study Design and Enterococcus Isolates

In this laboratory-based molecular diagnostic study, we evaluated 130 non-duplicate clinical Enterococcus isolates. The first set comprised 100 archived isolates from our previously published LAMP-LFD study [22], which were recovered from urine specimens processed at Nopparat Rajathanee Hospital, Bangkok, Thailand; 52 were *vanA*-positive, and 48 were *vanA*-negative, as determined by conventional PCR. An additional 30 non-duplicate isolates, not included in the earlier dataset, were recovered from clinical urine specimens processed at the same hospital between April and August 2026. This additional set comprised 13 vancomycin-resistant Enterococcus (VRE) and 17 vancomycin-susceptible Enterococcus (VSE) isolates, with one isolate retained per patient episode. Overall, the LAMP-HNB evaluation included 65 *vanA*-positive and 65 *vanA*-negative isolates, determined according to conventional PCR. The reused 100-isolate set enabled paired comparison with the previous LFD endpoint, whereas the 30 additional isolates extended evaluation of the HNB format.

### 4.2. Ethical Considerations

The study protocol was reviewed and approved by the Institutional Review Board of Rangsit University (approval no. RSUERB2026-015). In this study, we used anonymized Enterococcus isolates recovered from residual clinical urine specimens collected during routine diagnostic procedures at Nopparat Rajathanee Hospital, Bangkok, Thailand. All isolates and associated laboratory data were de-identified before inclusion, and no personally identifiable information was accessed or analyzed. The requirement for individual informed consent was waived because the study involved anonymized residual clinical materials and archived bacterial isolates and imposed no additional intervention or risk to patients.

### 4.3. Bacterial Identification and Phenotypic Characterization

Enterococcus isolates were identified using routine microbiological procedures at the Clinical Microbiology Laboratory of Nopparat Rajathanee Hospital, Bangkok, Thailand. The 100 archived isolates were identified by MALDI-TOF MS (Beckman Coulter, Inc., Brea, CA, USA) and confirmed by biochemical testing. Antimicrobial susceptibility to vancomycin was determined as described previously [22]. The 30 additional isolates were processed using the same laboratory’s routine identification and antimicrobial susceptibility testing procedures, with vancomycin susceptibility interpreted according to the CLSI2026 criteria applicable during the study period. Thirteen additional isolates were classified phenotypically as VRE and 17 as VSE. Conventional PCR was used as the molecular comparator for the presence or absence of *vanA*; therefore, assay performance reported here refers specifically to *vanA* detection rather than to all mechanisms of phenotypic vancomycin resistance. Bacterial isolates were initially cultured on blood agar plates and incubated aerobically at 37 °C for 24 h. Following incubation, colonies were harvested and subjected to genomic DNA extraction for subsequent LAMP-HNB and conventional PCR analyses.

### 4.4. Genomic DNA Extraction

Genomic DNA was extracted from bacterial colonies using the Presto Mini gDNA Bacterial Extraction Kit (Geneaid Biotech Ltd., New Taipei City, Taiwan) according to the manufacturer’s instructions. DNA concentration and purity were assessed using a NanoDrop 2000 spectrophotometer (Thermo Fisher Scientific, Waltham, MA, USA), and extracts were stored at −20 °C until analysis. *E. faecium* ATCC 700221 served as the *vanA*-positive control.

### 4.5. vanA Target and LAMP Primer System

The LAMP-HNB assay used the *vanA* primer set developed for the preceding LAMP-LFD platform and designed from GenBank sequence JN982328.1 [22]. The set comprised outer primers F3 and B3 and inner primers FIP and BIP, collectively recognizing six regions of the target (Table 8). Unlabeled primers were used for the HNB format because lateral flow tags were not required. All primers were synthesized by Pacific Science Co., Ltd. (Biobasic, Markham, ON, Canada).

### 4.6. Conventional PCR Comparator

Conventional PCR targeting *vanA* was performed using the F3/B3 primer pair. Each 25 µL reaction mixture contained 10× PCR buffer (Vivantis Technologies Sdn. Bhd., Selangor, Malaysia), 0.4 µM of each primer (F3, B3), 2.8 mM MgCl_2_ (Vivantis Technologies Sdn. Bhd., Selangor, Malaysia), 0.2 mM dNTPs (New England Biolabs, Ipswich, MA, USA), 2 U *Taq* DNA polymerase (Vivantis Technologies Sdn. Bhd., Selangor, Malaysia), 2 µL template DNA, and nuclease-free water was served as no template control. The *vanA E. faecium* ATCC 700221 DNA sample was used as a positive control. Conventional PCR was performed under the following cycling conditions: initial denaturation step of 5 min at 95 °C, followed by 35 cycles of 92 °C for 30 s where annealing temperature varied from 50 to 59 °C (1 °C interval) for 30 s and 72 °C for 1 min, and a final extension step at 72 °C for 5 min in a BIO-RAD T100 Thermal Cycler (Bio-Rad Laboratories, Inc., Hercules, CA USA). The expected 204 bp of PCR product was visualized by 2% agarose gel electrophoresis at 100 V for 30 min, stained with NEOgreen Gel Stain (NeoScience Co., Ltd., Suwon, Republic of Korea), and observed under a GelDoc Go Imaging System (Bio-Rad Laboratories, Hercules, CA, USA). 

The DNA concentration was measured using a NanoDrop spectrophotometer; the template DNA concentration was maintained at 50 ng. Because the conventional PCR and LAMP-HNB assays shared the same outer primers (F3/B3), conventional PCR was considered a non-independent molecular comparator rather than an independent reference standard.

### 4.7. LAMP Reaction and HNB Colorimetric Detection

The LAMP reaction was conducted in a 25 μL reaction mixture containing sterile water, 1× supplied buffer (New England Biolabs, Ipswich, MA, USA), 2.0 μM each of FIP and BIP primers, 0.2 μM each of F3 and B3 primers, 1.6 mM dNTPs (New England Biolabs, Ipswich, MA, USA), 0.5 M betaine (Sigma-Aldrich, Louis, MO, USA), 8 units of *Bst* 2.0 DNA polymerase (large fragment; New England Biolabs, Ipswich, MA, USA), and 2 µL of DNA template, In all experiments, the template DNA concentration was maintained at 50 ng. A 20 mM HNB stock solution was prepared in distilled deionized water. LAMP-HNB conditions were optimized sequentially by evaluating incubation temperatures of 61–65 °C; MgSO_4_ concentrations of 3.0–5.5 mM; HNB concentrations of 1.0, 2.5, 5.0, 7.5, and 10 μM; and reaction times of 15, 20, 25, 30, 45, and 60 min. A total of 1 µL HNB was incorporated into the reaction mixture before incubation. The LAMP-HNB assay was incubated in either a thermal cycler or a dry bath incubator (Hangzhou Allsheng Instruments Co., Ltd., Hangzhou, China), and nuclease-free water was used as a negative control to check for cross-contamination. The LAMP-HNB reactions were interpreted visually as positive and negative results based on appearing blue and violet, respectively, and the corresponding products from each optimization experiment were analyzed by 2% agarose gel electrophoresis to verify amplification at 100 volts for 30 min, then stained with NEOgreen Gel Stain (NeoScience Co., Ltd., Daejeon, South Korea). The LAMP products were then observed under a GelDoc Go Imaging System (Bio-Rad Laboratories, Hercules, CA, USA).

### 4.8. Analytical Specificity

Analytical specificity was evaluated using the same *vanA*-negative reference strain panel previously used for the LAMP-LFD assay [22]: *Staphylococcus aureus* ATCC 25923, oxacillin-resistant *S. aureus* ATCC 43300, *Enterococcus faecalis* ATCC 29212, *vanB*-harboring *E. faecalis* ATCC 51299, *Streptococcus pneumoniae* ATCC 49619, *Escherichia coli* ATCC 25922, *Acinetobacter lwoffii* ATCC 15309, *Enterobacter cloacae* ATCC 23355, carbapenem-resistant *Klebsiella pneumoniae* ATCC BAA-1705, *Klebsiella aerogenes* ATCC 13048, *Pseudomonas aeruginosa* ATCC 27853, and *Burkholderia cepacia* ATCC 25416. DNA from each strain was tested by LAMP-HNB under optimized conditions. Results were interpreted visually and confirmed by 2% agarose gel electrophoresis. *E. faecium* ATCC 700221 carrying the *vanA* gene and nuclease-free water served as positive and no-template controls, respectively.

All bacterial DNA for the analytical specificity test was extracted from bacterial colonies using the Presto Mini gDNA Bacterial Extraction Kit (Geneaid Biotech Ltd., New Taipei City, Taiwan) according to the manufacturer’s instructions. DNA concentration and purity were assessed using a NanoDrop 2000 spectrophotometer (Thermo Fisher Scientific, Waltham, MA, USA), and extracted DNA was stored at −20 °C until analysis. DNA concentrations and estimated genomic copy numbers of the bacterial reference strains used for LAMP-HNB assay analytical specificity evaluation are shown in Supplementary Table S1.

### 4.9. Analytical Sensitivity and Limit of Detection

Analytical sensitivity was determined using 10-fold serial dilutions of genomic DNA from *vanA*-positive *E. faecium* ATCC 700221, which ranged from 285.18 ng/µL to 2.852 fg/µL. Each dilution was tested in parallel by LAMP-HNB and conventional PCR in triplicate independent runs. For the former, a positive endpoint required concordance between the visual HNB color change and the characteristic amplification pattern on 2% agarose gel electrophoresis. PCR positivity was defined by detection of the expected *vanA*-specific amplicon. The LOD was the lowest DNA concentration that was positive in all three replicates. Detection endpoints of the two methods were then compared. Because the dilution interval was tenfold and only three replicate reactions were tested per concentration, the reported endpoint must not be interpreted as a statistically determined LOD95. Further twofold or fivefold dilutions and 20–30 replicates per concentration were not performed.

Estimated genomic DNA copy number calculation: Genomic DNA concentrations were measured using a NanoDrop spectrophotometer and converted into estimated genome copy numbers based on the molecular weight of double-stranded DNA and the corresponding bacterial genome size. The number of genome copies per microliter was calculated using the following equation:

Genome copies/µL = [DNA concentration (ng/µL) × 6.022 × 10^14^]/[Genome size (bp) × 660]

where 6.022 × 10^23^ represents Avogadro’s constant and 660 g/mol represents the average molecular weight of one base pair of double-stranded DNA [23,24,25].

For the analytical sensitivity evaluation, an approximate genome size of 3.0 Mb was assumed for *Enterococcus faecium*. For specificity, the genomic DNA concentrations of 12 bacterial reference strains were used to estimate genome copy numbers based on approximate genome sizes ranging from 2.1 to 8.0 Mb. The estimated number of genome copies per reaction was calculated by multiplying the genome copies per microliter by the DNA template volume added to each reaction.

These calculations represent theoretical genome equivalents and do not constitute direct quantification of *vanA* gene copies.

### 4.10. Application of LAMP-HNB to the 130-Isolate Collection

DNA from all 130 isolates was tested by conventional PCR and LAMP-HNB and classified as *vanA*-positive or *vanA*-negative. Analyses were performed for the 100-isolate overlapping cohort, the 30-isolate additional cohort, and the combined dataset. Results for the overlapping cohort were also compared with the previously reported LAMP-LFD classifications [22]. In this study, *vanA*-positive and *vanA*-negative refer specifically to the presence or absence of a *vanA* amplicon (expected 204 bp) as determined by the F3/B3 conventional PCR comparator; these categories do not establish the absence of other vancomycin resistance genes, such as *vanB*, in the clinical isolates.

### 4.11. Statistical Analysis

Conventional PCR was treated as the molecular comparator for *vanA* detection. A 2 × 2 contingency table was used to determine true-positive, true-negative, false-positive, and false-negative results. Sensitivity was calculated as TP/(TP + FN), specificity as TN/(TN + FP), PPV as TP/(TP + FP), NPV as TN/(TN + FN), and overall agreement/accuracy as (TP + TN)/N. Exact two-sided 95% confidence intervals for binomial proportions were calculated using the Clopper–Pearson method, and agreement beyond chance was summarized using Cohen’s kappa. Because this study’s collection was balanced by design rather than sampled according to clinical prevalence, PPV and NPV were interpreted as properties of the tested dataset and not population prevalence estimates.

## 5. Conclusions

Under optimized conditions (63 °C for 45 min with 4.0 mM MgSO_4_ and 5 μM HNB), LAMP-HNB showed complete agreement with conventional PCR for *vanA* detection in the evaluated 130-isolate collection and reached a 10-fold lower DNA concentration endpoint in the analytical dilution series. In the 100 overlapping isolates, LAMP-HNB preserved the classifications previously obtained with LAMP-LFD, indicating comparable classification in that set while offering a simpler closed-tube endpoint. The 30 additional isolates provided supportive, but limited, evidence beyond the original cohort. Independent prospective multicenter validation, broader genotype coverage, and formal assessment of visual-readout reproducibility are required before routine clinical implementation.

## Figures and Tables

**Table 1:** Composition of the Enterococcus isolate dataset used to evaluate LAMP-HNB.

Dataset Component	*vanA*-Positive by PCR	*vanA*-Negative by PCR	Total	Status
Previously characterized LAMP-LFD cohort	52	48	100	Previously published biological cohort; retested by HNB
Additional LAMP-HNB isolates	13	17	30	Additional isolates for the present study
Overall LAMP-HNB evaluation	65	65	130	Combined analysis

**Table 2:** Optimization of reaction conditions for the *vanA* LAMP-HNB assay.

Parameter	Conditions Evaluated	Selected Condition	Confirmation
Incubation temperature	61–65 °C	63 °C	Clear HNB endpoint; LAMP ladder on 2% AGE
MgSO_4_ concentration	3.0–5.5 mM	4.0 mM	Clear color discrimination; amplification confirmed by 2% AGE
HNB concentration	1.0, 2.5, 5.0, 7.5, 10 μM	5 μM	Clearest violet-to-blue discrimination; concordant AGE
Reaction time	15, 20, 25, 30, 45, 60 min	45 min	Clear endpoint at 45 min; comparable to 60 min; LAMP products confirmed by 2% AGE
Final optimized condition	—	63 °C; 4.0 mM MgSO_4_; 5 μM HNB; 45 min	Used for subsequent assay evaluation

**Table 3:** Two-by-two comparison of LAMP-HNB with conventional PCR for *vanA* detection in all 130 Enterococcus isolates.

LAMP-HNB Result	PCR-Positive	PCR-Negative	Total
Positive	65	0	65
Negative	0	65	65
Total	65	65	130

**Table 4:** Diagnostic performance of LAMP-HNB relative to conventional PCR.

Parameter	Estimate	95% CI/Comment
Sensitivity	100% (65/65)	94.48–100%
Specificity	100% (65/65)	94.48–100%
PPV	100% (65/65)	Prevalence-dependent; balanced study set
NPV	100% (65/65)	Prevalence-dependent; balanced study set
Overall agreement/accuracy	100% (130/130)	97.20–100%
Cohen’s kappa	1.00	Perfect observed agreement

**Table 5:** Conversion of genomic DNA concentrations to estimated copy numbers for LAMP-HNB assay analytical sensitivity evaluation.

Dilution	DNA Concentration	Copies/µL	Copies/Reaction (2 µL)
10^−1^	28.52 ng/µL	8.67 × 10^6^	1.73 × 10^7^
10^−2^	2.852 ng/µL	8.67 × 10^5^	1.73 × 10^6^
10^−3^	285.2 pg/µL	8.67 × 10^4^	1.73 × 10^5^
10^−4^	28.52 pg/µL	8.67 × 10^3^	1.73 × 10^4^
10^−5^	2.852 pg/µL	8.67 × 10^2^	1.73 × 10^3^
10^−6^	285.2 fg/µL	8.67 × 10^1^	1.73 × 10^2^
10^−7^	28.52 fg/µL	8.67	17.35
10^−8^	2.852 fg/µL	0.867	1.735

**Table 6:** Analytical and operational comparison of conventional PCR, LAMP-HNB, and the previously reported LAMP-LFD platform.

Characteristic	Conventional PCR	LAMP-HNB (Current Study)	LAMP-LFD (Previous Study)
Amplification principle	Thermal cycling	Isothermal LAMP	Isothermal LAMP
Visual endpoint	Gel-based endpoint in this protocol	Pre-added HNB color change	Lateral flow test/control lines
Post-amplification tube opening	Yes, for gel analysis	No for visual HNB reading	Yes, for probe hybridization/LFD step
Additional endpoint consumables	Agarose gel/stain/imaging	HNB only	FITC probe, LFD strip, running buffer
LOD in the reported protocol	285.2 pg/µL, equivalent to approximately 8.67 × 10^4^ genome copies/µL	28.52 pg/µL, equivalent to approximately 8.67 × 10^3^ genome copies/µL	28.52 pg/µL [22]
100 overlapping isolates	52 positive/48 negative	52 positive/48 negative	52 positive/48 negative [22]
30 additional isolates	13 positive/17 negative	13 positive/17 negative	Not part of previous study
Overall current HNB dataset	Comparator: 65+/65−	65+/65−; 130/130 concordant	Not applicable to all 130

**Table 7:** Comparison of present study’s LAMP-HNB and the previously reported LAMP-HNB (Kim et al., 2014) [20].

Comparison	Kim et al. (2014) [20]	Present Study
Target gene	*vanA*	*vanA*
Amplification	LAMP	LAMP
Colorimetric indicator	HNB	HNB
Clinical isolates	56	130
Detection endpoint	80 pg DNA template	28.52 pg/µL (8.67 × 103 genome copies/µL)
Comparator	PCR	Conventional PCR
Main contribution	Demonstration of vanA LAMP-HNB	Additional evaluation in 130 clinical isolates

**Table 8:** List of LAMP primers for *Enterococcus faecium*’s *vanA* gene.

Primer Name	5′ > 3′ Oligonucleotide Sequences
F3-*VanA*	CGATATTCAAAGCTCAGCAAT
B3-*VanA*	CGCTATTGACTTTTTTCACAC
FIP-*VanA*	AAGGCGGGAGTAGCTATCCC-TTTTT-TTGTATGGACAAATCGTTGAC
BIP-*VanA*	CCGGTGGCAGCTACGTTTAC-TTTTT-CGAAGGATGAGCCTGAAC

## Data Availability

The data presented in this study are available within the article and its Supplementary Materials.

## Supplementary Materials

The following supporting information can be downloaded at https://www.mdpi.com/article/10.3390/ijms27219927/s1.

## Abbreviations

The following abbreviations are used in this manuscript:

AGE	agarose gel electrophoresis
AMR	antimicrobial resistance
AST	antimicrobial susceptibility testing
ATCC	American Type Culture Collection
CI	confidence interval
CLSI	Clinical and Laboratory Standards Institute
DDW	distilled deionized water
DNA	deoxyribonucleic acid
dNTP	deoxynucleoside triphosphate
HNB	hydroxy naphthol blue
LAMP	loop-mediated isothermal amplification
LFD	lateral flow dipstick
LOD	limit of detection
MIC	minimum inhibitory concentration
NPV	negative predictive value
NTC	no-template control
PCR	polymerase chain reaction
PPV	positive predictive value
VRE	vancomycin-resistant Enterococcus
VSE	vancomycin-susceptible Enterococcus

## References

[1] Arias C.A., Murray B.E. (2012). The rise of the Enterococcus: Beyond vancomycin resistance. Nat. Rev. Microbiol..

[2] Murray B.E. (2000). Vancomycin-resistant enterococcal infections. N. Engl. J. Med..

[3] Cetinkaya Y., Falk P., Mayhall C.G. (2000). Vancomycin-resistant enterococci. Clin. Microbiol. Rev..

[4] Ahmed M.O., Baptiste K.E. (2018). Vancomycin-resistant enterococci: A review of antimicrobial resistance mechanisms and perspectives of human and animal health. Microb. Drug Resist..

[5] World Health Organization (2024). WHO Bacterial Priority Pathogens List, 2024: Bacterial Pathogens of Public Health Importance to Guide Research, Development and Strategies to Prevent and Control Antimicrobial Resistance.

[6] Arthur M., Courvalin P. (1993). Genetics and mechanisms of glycopeptide resistance in enterococci. Antimicrob. Agents Chemother..

[7] Arthur M., Reynolds P.E., Depardieu F., Evers S., Dutka-Malen S., Quintiliani R., Courvalin P. (1996). Mechanisms of glycopeptide resistance in enterococci. J. Infect..

[8] Walsh C.T., Fisher S.L., Park I.S., Prahalad M., Wu Z. (1996). Bacterial resistance to vancomycin: Five genes and one missing hydrogen bond tell the story. Chem. Biol..

[9] Healy V.L., Lessard I.A.D., Roper D.I., Knox J.R., Walsh C.T. (2000). Vancomycin resistance in enterococci: Reprogramming of the D-Ala-D-Ala ligases in bacterial peptidoglycan biosynthesis. Chem. Biol..

[10] Leclercq R., Dutka-Malen S., Brisson-Noël A., Molinas C., Derlot E., Arthur M., Duval J., Courvalin P. (1992). Resistance of enterococci to aminoglycosides and glycopeptides. Clin. Infect. Dis..

[11] Dutka-Malen S., Evers S., Courvalin P. (1995). Detection of glycopeptide resistance genotypes and identification to the species level of clinically relevant enterococci by PCR. J. Clin. Microbiol..

[12] Notomi T., Okayama H., Masubuchi H., Yonekawa T., Watanabe K., Amino N., Hase T. (2000). Loop-mediated isothermal amplification of DNA. Nucleic Acids Res..

[13] Nagamine K., Hase T., Notomi T. (2002). Accelerated reaction by loop-mediated isothermal amplification using loop primers. Mol. Cell. Probes.

[14] Mori Y., Nagamine K., Tomita N., Notomi T. (2001). Detection of loop-mediated isothermal amplification reaction by turbidity derived from magnesium pyrophosphate formation. Biochem. Biophys. Res. Commun..

[15] Tomita N., Mori Y., Kanda H., Notomi T. (2008). Loop-mediated isothermal amplification of gene sequences and simple visual detection of products. Nat. Protoc..

[16] Parida M., Sannarangaiah S., Dash P.K., Rao P.V.L., Morita K. (2008). Loop mediated isothermal amplification (LAMP): A new generation of innovative gene amplification technique; perspectives in clinical diagnosis of infectious diseases. Rev. Med. Virol..

[17] Goto M., Honda E., Ogura A., Nomoto A., Hanaki K. (2009). Colorimetric detection of loop-mediated isothermal amplification reaction by using hydroxy naphthol blue. BioTechniques.

[18] Mori Y., Notomi T. (2009). Loop-mediated isothermal amplification (LAMP): A rapid, accurate, and cost-effective diagnostic method for infectious diseases. J. Infect. Chemother..

[19] Soroka M., Wasowicz B., Rymaszewska A. (2021). Loop-mediated isothermal amplification (LAMP): The better sibling of PCR?. Cells.

[20] Kim H.J., Kim Y.J., Yong D.E., Lee K., Park J.H., Lee J.M., Yoon S.S. (2014). Loop-mediated isothermal amplification of *vanA* gene enables a rapid and naked-eye detection of vancomycin-resistant enterococci infection. J. Microbiol. Methods.

[21] Huang Q.Q., Liu B.B., Zhu H.F., Ma J.J., Tsoi M., Yao B.Q., Yao L.C., Wu Q., Mu X.Q., Liu S.L. (2019). Rapid and sensitive detection of the *vanA* resistance gene from clinical *Enterococcus faecium* and *Enterococcus faecalis* isolates by loop-mediated isothermal amplification. J. Glob. Antimicrob. Resist..

[22] Phurijaruyangkun S., Tangjitrungrot P., Pongparit S., Wattanakul N., Jantrapanukorn B., Veeramano R., Augkarawaritsawong S., Kumkrong K., Sirothot T., Jaratsing P. (2026). Development of an assay involving loop-mediated isothermal amplification with a lateral flow dipstick for detection of the *vanA* gene in vancomycin-resistant enterococci. Int. J. Mol. Sci..

[23] Kitts P.A., Church D.M., Thibaud-Nissen F., Choi J., Hem V., Sapojnikov V., Smith R.G., Tatusova T., Xiang C., Zherikov A. (2016). Assembly: A resource for assembled genomes at NCBI. Nucleic Acids Res..

[24] Yarmosh D.A., Lopera J.G., Puthuveetil N.P., Combs P.F., Reese A.L., Tabron C., Pierola A.E., Duncan J., Greenfield S.R., Marlow R. (2022). Comparative Analysis and Data Provenance for 1113 Bacterial Genome Assemblies. mSphere.

[25] Minogue T.D., Daligault H.A., Davenport K.W., Bishop-Lilly K.A., Broomall S.M., Bruce D.C., Chain P.S., Chertkov O., Coyne S.R., Freitas T. (2014). Complete Genome Assembly of *Escherichia coli* ATCC 25922, a Serotype O6 Reference Strain. Genome Announc..

