# Supplementary material for: Colorimetric Detection of the vanA Gene in Vancomycin-Resistant Enterococcus Using Loop-Mediated Isothermal Amplification with Hydroxy Naphthol Blue
Source: Int J Mol Sci. 2026 Nov 5;27(21):9927. doi: 10.3390/ijms27219927 (PMC13764336; doi:10.3390/ijms27219927)
Supplement: Supplementary file 1 [file ijms-27-09927-s001.zip › ijms-4624062-supplementary.pdf]

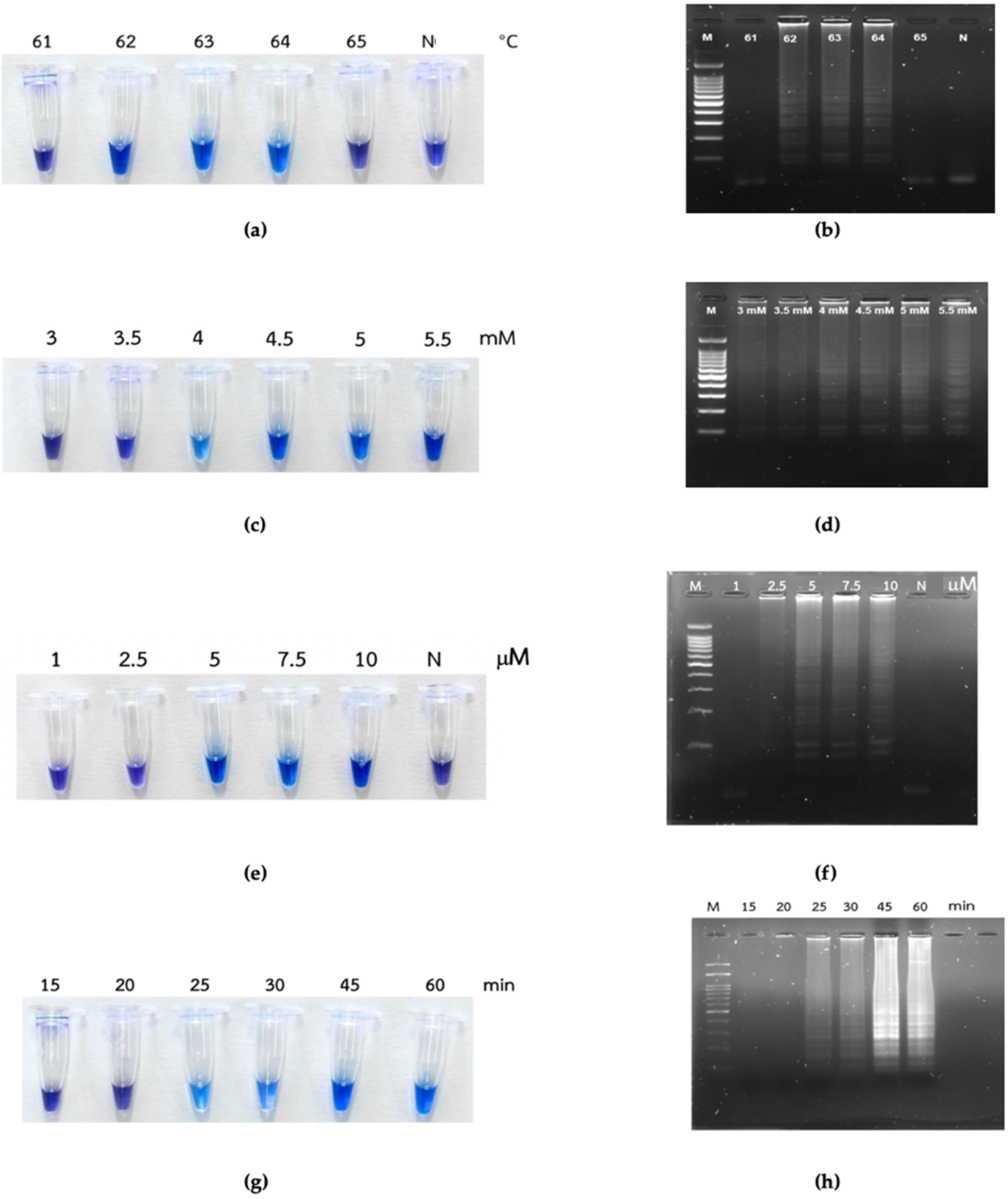

Figure S1. The optimization conditions of LAMP-HNB: (**a**) The optimization of thermal conditions between 61 - 65 ˚C. (**c**) The optimization of $MgSO_4$ in the range of 3–5.5 mM. (**e**) The optimization of HNB concentration in the range of 1-10 μM. (**g**) The optimal reaction durations were observed at 15 - 60 min. (b,d,f,h) All of the optimized conditions were confirmed by 2% AGE. Lane M represents Hyper Ladder 100 bp Plus (Bioline Reagents Ltd., London, UK), and N, no-template control.

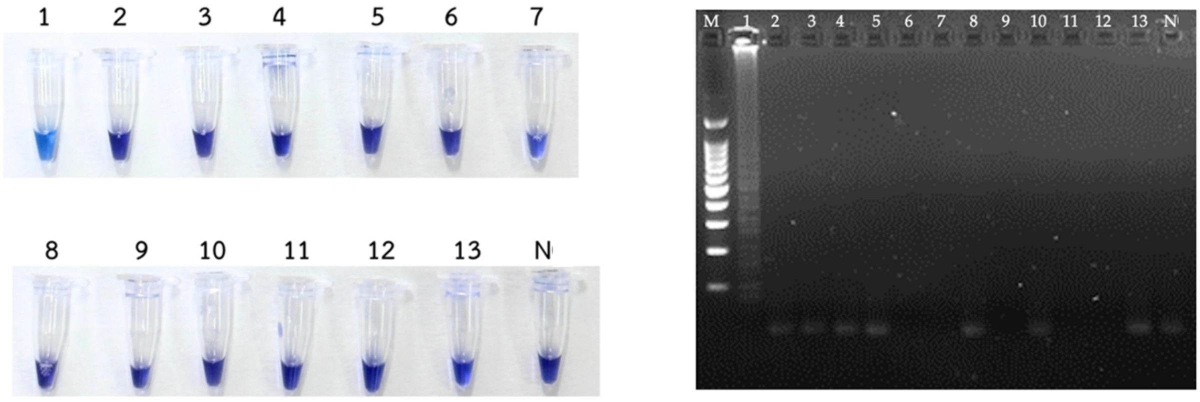

(a) (b)

Figure S2. Analytical specificity of the LAMP-HNB assay for detection of the *vanA* gene. (a) The analytical specificity of the LAMP-HNB assay was evaluated using: Lane 1, *vanA*-positive *Enterococcus faecium* ATCC 700221; and 12 non-target bacterial strains, including Lane 2, *Staphylococcus aureus* ATCC 25923; Lane 3, oxacillin-resistant *S. aureus* ATCC 43300; Lane 4, *Enterococcus faecalis* ATCC 29212; Lane 5, *vanB*-positive *E. faecalis* ATCC 51299; Lane 6, *Streptococcus pneumoniae* ATCC 49619; Lane 7, *Escherichia coli* ATCC 25922; Lane 8, *Acinetobacter lwoffii* ATCC 15309; Lane 9, *Enterobacter cloacae* ATCC 23355; Lane 10, carbapenem-resistant *Klebsiella pneumoniae* ATCC BAA-1705; Lane 11, *Klebsiella aerogenes* ATCC 13048; Lane 12, *Pseudomonas aeruginosa* ATCC 27853; Lane 13, *Burkholderia cepacia* ATCC 25416. Lane M represents Hyper Ladder 100 bp Plus (Bioline Reagents Ltd., London, UK), and N, no-template control. Only the *vanA*-positive reaction turned blue, whereas all non-target organisms and the no-template control remained violet, indicating no detectable cross-reactivity under the tested conditions. **(b)** The analytical specificity of the LAMP-HNB was confirmed by 2% AGE

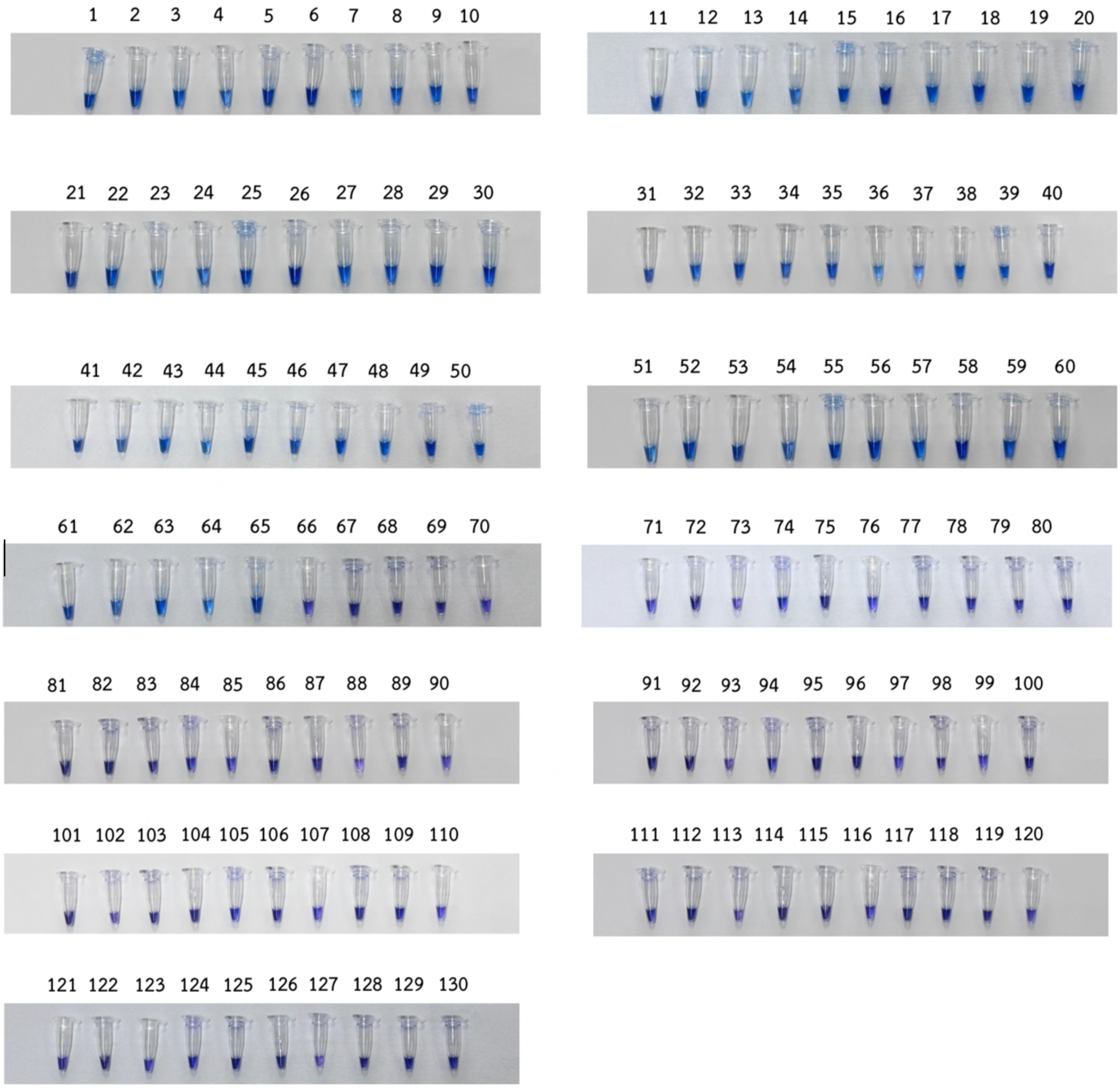

Figure S3. Visual detection of *vanA* by LAMP-HNB in 130 clinical *Enterococcus* isolates. Representative colorimetric results of the LAMP-HNB assay for *vanA* detection in 130 clinical *Enterococcus* isolates. Samples No. 1–65 represented positive LAMP-HNB reactions, indicated by a color change from violet to blue, whereas samples No. 66–130 represented negative reactions and retained the original violet color. Overall, LAMP-HNB classified 65 isolates as *vanA*-positive and 65 as *vanA*-negative. The LAMP-HNB results showed 100% concordance with conventional PCR, with no discordant results observed among the 130 isolates.

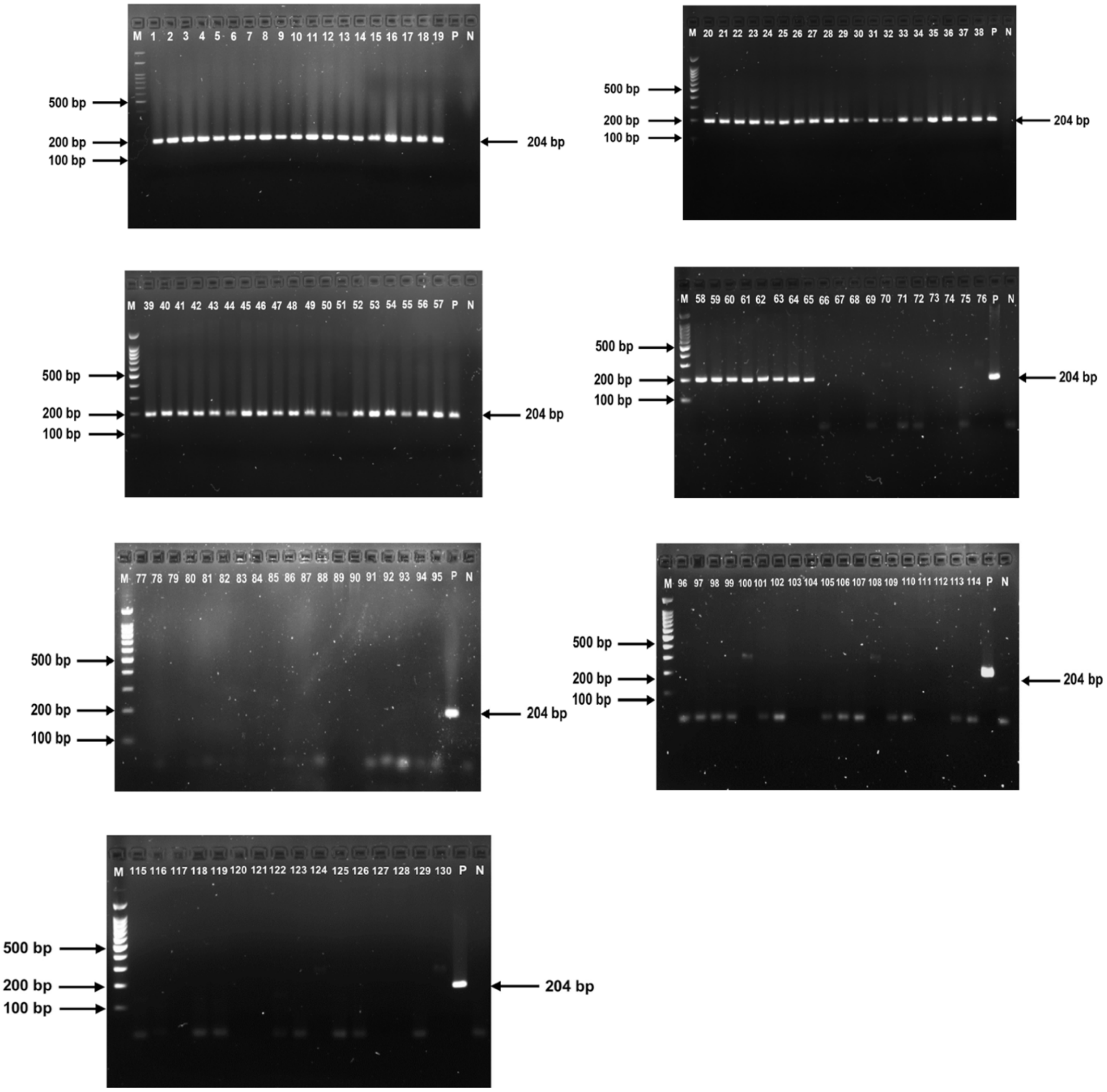

Figure S4. Conventional PCR detection of the *vanA* gene in 130 clinical *Enterococcus* isolates. Representative agarose gel electrophoresis results of conventional PCR for detection of the *vanA* gene in 130 clinical *Enterococcus* isolates. Samples No. 1–65 were positive for *vanA*, as demonstrated by the presence of the expected 204-bp amplicon, whereas samples No. 66–130 were negative, with no target-specific amplification observed. Overall, conventional PCR identified 65 *vanA*-positive and 65 *vanA*-negative isolates. The PCR results showed 100% concordance with the LAMP-HNB assay (130/130 isolates), with no discordant results observed. M represents Hyper Ladder 100 bp Plus (Bioline Reagents Ltd., London, UK); P, positive control; N, no-template control.

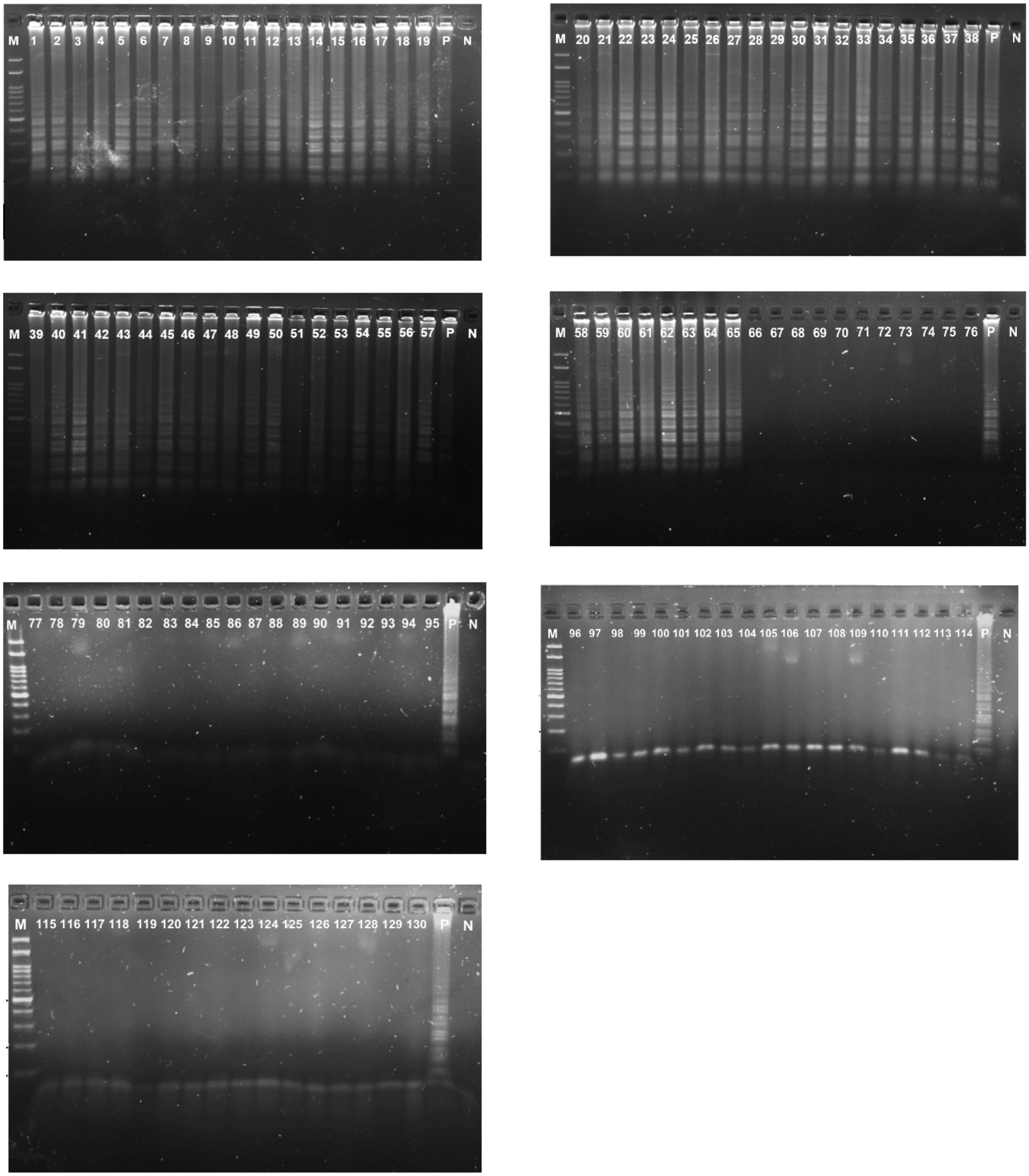

Figure S5. Agarose gel electrophoresis analysis of LAMP products for *vanA* detection in 130 clinical *Enterococcus* isolates. Representative 2% agarose gel electrophoresis results of LAMP amplification targeting the *vanA* gene. Samples No. 1–65 showed positive LAMP amplification, characterized by the typical ladder-like pattern of multiple DNA bands, whereas samples No. 66–130 showed no detectable amplification and were classified as negative. M represents Hyper Ladder 100 bp Plus (Bioline Reagents Ltd., London, UK); P**,** positive control; N, no-template control. Overall, LAMP identified 65 *vanA*-positive and 65 *vanA*-negative isolates. The LAMP results showed 100% concordance with conventional PCR (130/130 isolates), with no discordant results observed.

Table S1. DNA concentrations and estimated genomic copy numbers of bacterial reference strains used for analytical specificity evaluation of the LAMP-HNB assay.

Bacterial reference strain	DNA (ng/μL)	Genome size (Mb)*	Copies/μL	Copies/reaction (2 μL)
Staphylococcus aureus ATCC 25923	228.45	2.8	7.44×10^7	1.49×10^8
Oxacillin-resistant *Staphylococcus aureus* ATCC 43300	246.41	2.8	8.03×10^7	1.61×10^8
Enterococcus faecalis ATCC 29212	279.68	3.2	7.97×10^7	1.59×10^8
vanB-harboring *Enterococcus faecalis* ATCC 51299	238.88	3.2	6.81×10^7	1.36×10^8
Streptococcus pneumoniae ATCC 49619	198.76	2.1	8.64×10^7	1.73×10^8
Escherichia coli ATCC 25922	289.95	5.1	5.19×10^7	1.04×10^8
Acinetobacter lwoffii ATCC 15309	269.78	3.5	7.03×10^7	1.41×10^8
Enterobacter cloacae ATCC 23355	232.56	5.0	4.24×10^7	8.49×10^7
Carbapenem-resistant *Klebsiella pneumoniae* ATCC BAA-1705	215.57	5.5	3.58×10^7	7.15×10^7
Klebsiella aerogenes ATCC 13048	199.87	5.3	3.44×10^7	6.88×10^7
Pseudomonas aeruginosa ATCC 27853	206.76	6.3	2.99×10^7	5.99×10^7
Burkholderia cepacia ATCC 25416	214.72	8.0	2.45×10^7	4.90×10^7

*Genome sizes are approximate representative values, not verified strain-specific genome lengths. All copy numbers are theoretical estimates calculated from the supplied DNA concentrations.
